# Fabric Classification Using a Finger-Shaped Tactile Sensor *via* Robotic Sliding

**DOI:** 10.3389/fnbot.2022.808222

**Published:** 2022-02-23

**Authors:** Si-ao Wang, Alessandro Albini, Perla Maiolino, Fulvio Mastrogiovanni, Giorgio Cannata

**Affiliations:** ^1^MACLAB, Dipartimento di Informatica, Bioingegneria, Robotica e Ingegneria dei Sistemi, Università degli Studi di Genova, Genoa, Italy; ^2^Oxford Robotics Institute, University of Oxford, Oxford, United Kingdom; ^3^TheEngineRoom, Dipartimento di Informatica, Bioingegneria, Robotica e Ingegneria dei Sistemi, Università degli Studi di Genova, Genoa, Italy

**Keywords:** active touching, robotic touch, tactile sensing, texture identification, haptic perception

## Abstract

Tactile sensing endows the robots to perceive certain physical properties of the object in contact. Robots with tactile perception can classify textures by touching. Interestingly, textures of fine micro-geometry beyond the nominal resolution of the tactile sensors can also be identified through exploratory robotic movements like sliding. To study the problem of fine texture classification, we design a robotic sliding experiment using a finger-shaped multi-channel capacitive tactile sensor. A feature extraction process is presented to encode the acquired tactile signals (in the form of time series) into a low dimensional (≤7D) feature vector. The feature vector captures the frequency signature of a fabric texture such that fabrics can be classified directly. The experiment includes multiple combinations of sliding parameters, i.e., speed and pressure, to investigate the correlation between sliding parameters and the generated feature space. Results show that changing the contact pressure can greatly affect the significance of the extracted feature vectors. Instead, variation of sliding speed shows no apparent effects. In summary, this paper presents a study of texture classification on fabrics by training a simple k-NN classifier, using only one modality and one type of exploratory motion (sliding). The classification accuracy can reach up to 96%. The analysis of the feature space also implies a potential parametric representation of textures for tactile perception, which could be used for the adaption of motion to reach better classification performance.

## 1. Introduction

### 1.1. Tactile Sensing and Perception

Tactile sensing is fundamental for robots to understand the space surroundings by revealing some contact features not directly accessible to visual and acoustic sensors, including pressure, vibration, and temperature. Tactile sensors are specifically designed to convert the instant changes of these physical properties into electrical signals. Unlike visual and acoustic sensing, tactile sensing involves and probably only involves the direct mechanical interaction between the sensor and the object in contact with. In the majority of the cases, external environmental conditions like illumination, acoustic noise, humidity, and temperature do not affect the capability of tactile sensing. Despite its robust performance in different scenarios, tactile sensing is a very limited instrumental modality that only captures the regional stimulus around a sensor. Luckily, the limitation can be alleviated by pairing the sensing with exploratory robotic motions to enlarge the contact area, requiring both spatial and temporal decoding to interpret the signals. The process of decoding signals to comprehend the space surroundings is the core of tactile perception. Unfortunately, at the current stage, there is no such a uniform and standard format of tactile sensor and tactile data and hence tactile perception is tightly bonded to the specific sensing technology being used (Luo et al., 2017).

With the advancement in interactive control for robotics, tactile sensing is gaining attention in recent decades. Since robotic tasks with physical contacts are very likely to introduce visual occlusion, more studies using tactile sensing to perceive object shape/textures (Kaboli and Cheng, 2018; Kerr et al., 2018; Martinez-Hernandez et al., 2020; Fang et al., 2021) and executing dexterous manipulation (Jiménez, 2017; Belousov et al., 2019) are popping up. Results show that tactile sensing has great potential, especially in handling soft materials like fabrics considering its instant response to tiny variations of stimulus.

### 1.2. Fabric Classification *via* Active Perception

A better understanding of the objects that the robot is interacting with helps to adjust the control strategy and control parameters, leading to more efficient and possibly safer motions. Among all objects for interaction, fabrics are of particular interest to us as they are not only one of the most common soft materials in daily life but also intrinsically difficult to distinguish. Fabrics can be dyed into different colors so that vision alone has difficulty in identifying them. Textures of fabrics vary a lot and are usually so fine and complex that sometimes even human beings can barely distinguish (e.g., canvas, denim, and linen) with non-destructive methods.

To classify fabrics, usually, active motions are necessary to acquire a holistic tactile sample of the texture since tactile sensors only capture regional stimuli. Manfredi et al. (2014) found that the vibrations elicited during the interaction carry information about the microgeometry of the fabric surface and mechanical properties of the tactile sensor itself. In Fishel and Loeb (2012); Khan et al. (2016); Kaboli and Cheng (2018); Kerr et al. (2018) sliding motions are conducted in different manners to collect vibration signals about the fabric textures. Particularly, Fishel and Loeb (2012) show that changing exploratory actions can affect the received tactile signals and lead to different classification performances. However, how changing the motion parameters can affect the performance of the perception and fabric classification has not been thoroughly investigated.

### 1.3. Goals

Since tactile decoding and perception are tightly bonded with the specific sensors being used, one of the very first objectives is to construct a robust perception system that could extract certain tactile features from the tactile signals. The tactile features are desired to embed some peculiar information to the fabrics, independent of the variation of sliding parameters during the acquisition stage. The study Weber et al. (2013) on the tactile perception of human beings indicates that certain invariant tactile features can be retrieved by touching and sliding/rubbing. Our research serves to verify the feasibility of a similar idea on a robotic system.

In reality, it is ideal to have the capability of adapting the robotic behavior to compensate for the limitation of the sensing technology (e.g., bandwidth, resolution, geometric structures) and the perception algorithm since the mechatronic system itself is usually unmodifiable. Adaptation first requires an overall understanding of correlations between motions and tactile signals. Our study aims to give a general picture so that in the task of fabric classification *via* sliding, performance can be improved by simply adjusting the motion parameters.

We will also testify to the expansibility and scalability of the algorithm. Expansibility suggests that the algorithm applies to textures other than fabrics on the classification task, while scalability means that the system can incorporate tactile information from new fabrics in an iterative approach.

Beyond the classification task, another purpose of the research is to search for a potential parametric representation of the textures in the feature space that can be used further in a more complex system for fabric handling and manipulation.

### 1.4. Outline

Inspired by how humans try to identify fabrics with their skin solely, *via* sliding and rubbing fingers on the fabric surface, we command a robotic arm equipped with a capacitive tactile sensor on its end-effector to grip the fabric and slide. Different from some existing attempts of texture identification in Fishel and Loeb (2012); Khan et al. (2016); Kerr et al. (2018) that fix the inspectee material on a motorized platform where the sensor is stationary, our vibration signals are acquired during a dynamic process where fabric stripes are free to stretch and bend. We allow the 7-DOF robotic arm to carry out the exploratory motions in a large area, similar to the experiments in Bauml and Tulbure (2019) and Taunyazov et al. (2019), which resembles the daily scenario where humans touch to feel the fabrics.

Unlike Fishel and Loeb (2012); Khan et al. (2016) innovating on their features according to either physics or statistics, our algorithm seeks emerging frequency features by using an incremental principal component analysis (IPCA) method. It requires very little data for bootstrap compared to more complex neural network approaches and the explainability can be easily represented by the ratio of variance.

Our methods are tested upon a specific capacitive tactile sensor, but the algorithm per se is generic and applies to any mono-modality multi-channel tactile sensor to extract frequency features. With the extracted features, fabric textures can be classified by training a simple k-NN classifier.

We show that the proposed method is capable of decoding tactile signals and classifying the fabrics under different sliding pressures and speed settings. Very few frequency features suffice to represent the perceived fabric textures. An incremental IPCA method is applied to allow for iterative update of the feature extractor so that tactile information of new fabrics can be fused to improve the classification performance. Results imply that the distinguishability of fabrics not only depends on their microgeometry textures but also the physical properties including elasticity and friction coefficient that can only be perceived during a dynamic interaction. In the cases of ambiguity to classify certain fabrics, it is possible to increase the confidence and accuracy by adjusting the sliding speed and pressure to an optimal setting specific to those fabrics.

## 2. Related Works

The problem of discriminating textured objects or materials with the support of tactile sensing has been widely investigated in the literature. Most of the previous works integrate tactile sensors into robot end-effectors which are controlled to interact with the objects of interest. Tactile data collected during the interaction are then processed to extract features for texture classification using machine learning techniques.

The type of features extracted from tactile data usually depends on the sensing technology adopted. There are two major trends of methods in the task of texture classification. The first either employs a high-resolution vision-based sensor (Li and Adelson, 2013; Luo et al., 2018; Yuan et al., 2018) or crops the time-series data (Taunyazov et al., 2019) to construct tactile images and directly encode the spatial textures by neural networks (NNs). While the second type of method collects the tactile signals using sensors sensitive to vibrations. Tactile signals are first transformed into the frequency domain and then both temporal and frequency features are extracted to identify textures as in Fishel and Loeb (2012); Khan et al. (2016); Kerr et al. (2018); Massalim et al. (2020).

### 2.1. Spatial Features as Images

Li and Adelson (2013) directly use a vision-based GelSight sensor to classify 40 different materials. The high-resolution tactile image generated by the sensor captures geometric information on the texture of the specific material. In particular, the authors proposed a novel operator, the Multi Local Binary Patterns, taking both micro and macro structures of the texture into account for feature extraction.

Instead of classifying the exact type of material, the work proposed by Yuan et al. (2018) aims at recognizing 11 different properties from 153 varied pieces of clothes using a convolutional neural network (CNN) based architecture. Those properties are both physical (softness, thickness, durability, etc.) and semantic (e.g., washing method and wearing season). Moreover, a Kinect RGB-D camera is also used to help explore the clothes autonomously. The results showed great potential in the application of domestic help for clothes management.

Alternatively, Taunyazov et al. (2019) proposed an interaction strategy alternating static touches and sliding movements with controlled force, exploring the possibility to extract spatial features from a capacitive sensor using a CNN-LSTM (long-short-term memory) architecture. Experiments are performed on 23 materials using a capacitor-based skin covered on the iCub forearm, reaching 98% classification accuracy. Capacitive tactile sensors are usually more suitable for dexterous manipulations compared to vision-based sensors due to their compact sizes and less deformable contact surfaces. The possibility to apply a vision-based tactile perception method eases the usage of capacitive sensors.

Bauml and Tulbure (2019) presented another interesting research in this category. The proposed method makes use of the trendy transfer learning techniques to enable n-shot learning for the task of texture classification. The capability of learning from very few samples by taking advantage of a pre-trained dataset can be very handy for deploying tactile sensing systems on new robotic systems.

### 2.2. Temporal and Frequency Features

Fishel and Loeb (2012) conducted comprehensive research on texture classification using BioTac. Unlike most of the other works, their features are computed with specific physical meanings as traction, roughness, and fineness. Several combinations of sliding speeds and normal forces are also tested to enable a Bayesian inference.

Khan et al. (2016) described a similar experiment with hand-crafted statistical features to identify textures. The research employs a custom finger-shaped capacitive tactile sensor, which is mounted on the probe of a 5-axes machine and controlled to slide on a platform covered with the fabric. Both applied pressure and velocity are controlled for the sliding motions. The statistical features, computed both in frequency and time domains, are used to train a support-vector-machine (SVM) classifier to discriminate 17 different fabrics.

Another similar work is followed by Kerr et al. (2018) where PCA based feature extraction is performed on the tactile data. Both pressing and sliding motions are applied to acquire data and several different classifiers are evaluated.

A recent work Massalim et al. (2020) tries to not only identify textures but also detect slip and estimate the speed of sliding, using an accelerometer installed on the fingertips of the robotic gripper to record vibration. This work combined multiple deep learning techniques to achieve a decent classification accuracy.

### 2.3. Summary

Compared to some of the literature, our work differs mostly in two aspects:

1. The design of the experiments simulate a realistic application scenario where very few constraints are applied on the fabrics and the robotic sliding.
2. The perception system is very lightweight computationally, which can be implemented on a modern quad-core consumer PC; it tries to extract some intrinsic frequency features without the necessity to train on a large dataset (like other deep learning techniques) and the quality of these features are self-explanatory.

## 3. Methods

This section details the signal decoding and perception algorithms. We describe the pipeline of signal processing and feature extraction that maps the original tactile signals in large matrix form to low dimensional vectors and introduce a weighted k-NN classifier to identify the fabrics in the feature space.

A tactile sensor usually consists of several *taxels* (the minimal tactile sensing unit like pixels for cameras) that only perceive local stimuli and generate multi-channel signals over time. We follow a feature extraction method based on incremental principal component analysis (IPCA) to gradually extract the frequency features during the process of sliding and touching different types of fabrics. The feature extractor first transforms a tactile time series into a multi-channel frequency spectrum in the format of matrix and resamples the frequency spectrum to a fixed size. After that, the frequency spectrum (as a matrix) is *vectorized* (flattened). After collecting multiple tactile measurements and transforming them all to resampled, vectorized frequency spectra, we stack them together to form a large data matrix. Then, we apply IPCA transformer to project the data matrix to lower-dimensional vectors. With the condensed representation of tactile measurements, it is possible to classify fabric textures by training a k-nearest neighbors (k-NN) classifier.

### 3.1. Signal Processing

A tactile measurement, M-channel time series *X*, represented in the matrix form


(1) $$\begin{array}{l} X=\left[\begin{array}{cccc} x_{1}\left(0\right) & x_{2}\left(0\right) & \ldots & x_{M}\left(0\right)\\ \vdots & \vdots & \ldots & \vdots \\ x_{1}\left(t\right) & x_{2}\left(t\right) & \ldots & x_{M}\left(t\right)\\ \vdots & \vdots & \ldots & \vdots \\ x_{1}\left(N-1\right) & x_{2}\left(N-1\right) & \ldots & x_{M}\left(N-1\right) \end{array}\right]\in {\mathbb{R}}^{N\times M} \end{array}$$

is first normalized to


(2) $$\begin{array}{l} \hat{X}=\left[\begin{array}{cccc} \frac{x_{1}\left(0\right)-{\overset{-}{x}}_{1}}{\sigma_{1}} & \frac{x_{2}\left(0\right)-{\overset{-}{x}}_{2}}{\sigma_{2}} & \ldots & \frac{x_{M}\left(0\right)-{\overset{-}{x}}_{M}}{\sigma_{M}}\\ \vdots & \vdots & ... & \vdots \\ \frac{x_{1}\left(N-1\right)-{\overset{-}{x}}_{1}}{\sigma_{1}} & \frac{x_{2}\left(N-1\right)-{\overset{-}{x}}_{2}}{\sigma_{2}} & \ldots & \frac{x_{M}\left(N-1\right)-{\overset{-}{x}}_{M}}{\sigma_{M}} \end{array}\right]\in {\mathbb{R}}^{N\times M} \end{array}$$

using the channel mean and SD $\overset{-}{X}=\left[{\overset{-}{x}}_{1}\;{\overset{-}{x}}_{2}\;\ldots \;{\overset{-}{x}}_{M}\right]\in {\mathbb{R}}^{M}$ and $\sigma =\left[\sigma_{1}\;\sigma_{2}\;\ldots \;\sigma_{M}\right]\in {\mathbb{R}}^{M}$. Normalization brings the sensor signals acquired with different *Pressure* settings into the same scope such that comparative analyses are directly available. The mean-deviated and scaled tactile measurement is then transformed into the frequency domain by applying Fourier transform channel-wise, taking only the magnitude to gain the real frequency-spectra matrix *Y* defined by


(3) $$\begin{array}{l} Y=\left[\begin{array}{cccc} y_{1\left(0\right)} & y_{2\left(0\right)} & \ldots & y_{M\left(0\right)}\\ \vdots & \vdots & \ldots & \vdots \\ y_{1\left(N-1\right)} & y_{2\left(N-1\right)} & \ldots & y_{M\left(N-1\right)} \end{array}\right]\in {\mathbb{R}}^{N\times M} \end{array}$$

where each entry *y*_*a*(*b*)_ is given by


(4) $$\begin{array}{l} y_{a\left(b\right)}=\|\overset{N-1}{\underset{n=0}{\sum }}\frac{x_{a}\left(n\right)-{\overset{-}{x}}_{a}}{\sigma_{a}}e^{-i2\pi b\frac{n}{N}}\|. \end{array}$$

Resampling is necessary here to unify the sizes of different spectra as the scopes of the frequency spectra are dependent on the length of the original time series, which can vary among measurements since the experiments are conducted with several different sliding speeds. The re-sampled frequency matrix $Y\in {\mathbb{R}}^{N_{r}\times M}$, where *N*_*r*_ is a predefined resolution constant, is then *vectorized* (flattened) into a frequency-spectra vector $\vec{y}\in {\mathbb{R}}^{N_{r}M}$.

Multiple tactile measurements acquired in sliding motions, as vectors of the same dimension now, can be stacked together to form a new observation matrix


(5) $$\begin{array}{l} O=\left[\vec{y_{1}}\;\vec{y_{2}}\;...\;\vec{y_{K}}\right]\in {\mathbb{R}}^{N_{r}M\times K} \end{array}$$

containing all the frequency vectors, where *K* is the total number of measurements. We then resort to principal component analysis (PCA) on the observation matrix *O* for dimensionality reduction and feature extraction.

### 3.2. Feature Extraction

Incremental principal component analysis as an unsupervised method is well suitable for dimensionality reduction in our problem. It preserves as much as possible information contained in the original data matrices by minimizing a reconstruction loss. We apply the IPCA introduced in Ross et al. (2008) to fit the training dataset *O*.

Denoting the mean-deviated form of *O* as Ô. The goal is to find a feature matrix *Q* ∈ ℝ^*D*×*K*^ with *D* ≪ *N*_*r*_*M* such that the total reconstruction error


(6) $$\begin{array}{l} {\left\|Ô-\Phi Q\right\|}_{F} \end{array}$$

is minimized. Frobenius norm is chosen considering its fast and easy computation, while other similar matrix norms also function the same for this optimization setup. Here, $\Phi \in {\mathbb{R}}^{N_{r}M\times D}$ is a projection matrix mapping a frequency-spectra vector $\vec{y}\in {\mathbb{R}}^{N_{r}M}$ to a new feature vector $\vec{q}\in {\mathbb{R}}^{D}$.

Given *n* new measurements pre-processed and vectorized, formatted as a matrix


(7) $$\begin{array}{l} A=\left[\vec{y_{K+1}}\;\vec{y_{K+2}}\;\ldots \;\vec{y_{K+n}}\right] \end{array}$$

A brutal update for these *n* new data requires the computation of singular-value-decomposition (SVD) for the mean-deviated form of the augmented data matrix *O*_*K*+*n*_ = [*O A*], which is not ideal for online applications. In the presence of more tactile measurements, the data matrix keeps expanding, and traditional PCA will slow down drastically.

Incremental principal component analysis differs from traditional PCA in handling new data. Instead of re-computing the SVD for the entire augmented data matrix


(8) $$\begin{array}{l} {Ô}_{K+n}=\left[Ô\;Â\right] \end{array}$$

where Â is the mean-deviated form of *A*, only the SVD of the horizontal concatenation of the original and the additional data matrix, and one additional vector $\sqrt{\frac{Kn}{K+n}}\left(Ō-Ā\right)$ are needed. To obtain the SVD for the augmented data matrix Ô_*K*+*n*_, first we define


(9) $$\begin{array}{l} B=\left[Ā\;\sqrt{\frac{Kn}{K+n}}\left(Ō-Ā\right)\right] \end{array}$$

and compute


(10) $$\begin{array}{l} \tilde{B}=\text{orth}\left(B-UU^{T}B\right) \end{array}$$

where **orth** performs orthogonalization and


(11) $$\begin{array}{l} R=\left(\begin{array}{cc} \Sigma & U^{T}B\\ 0 & \tilde{B}\left(B-UU^{T}B\right) \end{array}\right) \end{array}$$

*via* QR decomposition $\left[U\;\tilde{B}\right]R\overset{QR}{=}\left[U\Sigma A\right]$. Then, we apply SVD to *R* as $R\overset{SVD}{=}Ũ\tilde{\Sigma }{Ṽ}^{T}$ and finally the equivalent SVD of ${Ô}_{K+n}=U^{\prime }\Sigma^{\prime }{V\prime }^{T}$ is given by $U^{\prime }=\left[U\tilde{B}\right]Ũ$ and $\Sigma^{\prime }=\tilde{\Sigma }$; whereas *V*′ is not directly used in IPCA, it is not calculated explicitly.

Considering that tactile measurements are acquired incrementally, the feature extractor can be trained upon known data. During the procedure where more new tactile measurements are presented the IPCA based feature extractor can first map the new data matrices to feature vectors to perform classification, and then partially fit the newly sampled data to incorporate the information and improve the performance. The application of the incremental method enables the feature extractor to adapt to the growing database fast and efficiently.

### 3.3. Identification and Classification

In the lower dimensional feature space, a weighted k-NN classifier (Dudani, 1976) can be fitted upon the training dataset. The trained classifier predicts the label of a query point in the *D*-dimensional feature space using distance-weighted voting by its *k* nearest points.

Given a feature vector $\vec{q^{\prime }}$ representing a new tactile measurement mapped in the feature space, to predict its label with all the other points, we define 𝔑 as the set of *k* nearest points to the query point $\vec{q^{\prime }}$ and compute


(12) $$\begin{array}{l} y^{\prime }=\underset{v}{arg max}\underset{\left({\text{q}}_{i},\vec{y_{i}}\right)\in 𝔑}{\sum }w_{i}I\left(v=\vec{y_{i}}\right) \end{array}$$

where $w_{i}=\frac{1}{d\left({\text{q}}^{\prime },{\text{q}}_{i}\right)}$ and *I* is the indicator function


$$I\left(v=y_{i}\right)=\{\begin{array}{cc} 1 & \text{if }{\text{q}}_{i}\text{ belongs to class }i\\ 0 & \text{otherwise} \end{array}$$

## 4. Experimental Setup

### 4.1. CPM-Finger Capacitive Tactile Sensor

**Figure 1:**
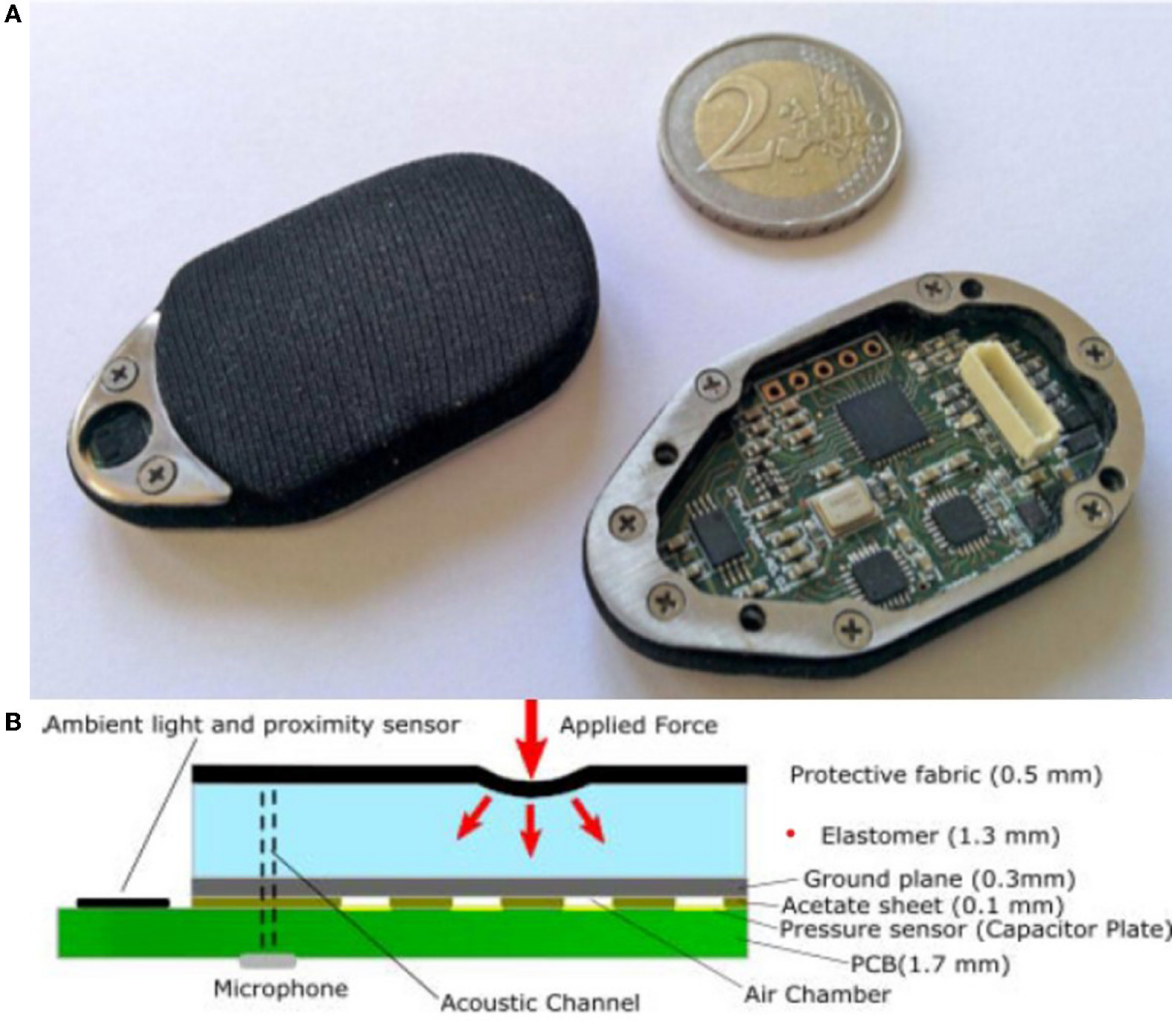
**(A,B)** Illustration of CPM-Finger tactile sensor (Denei et al., 2017).

**Figure 2:**
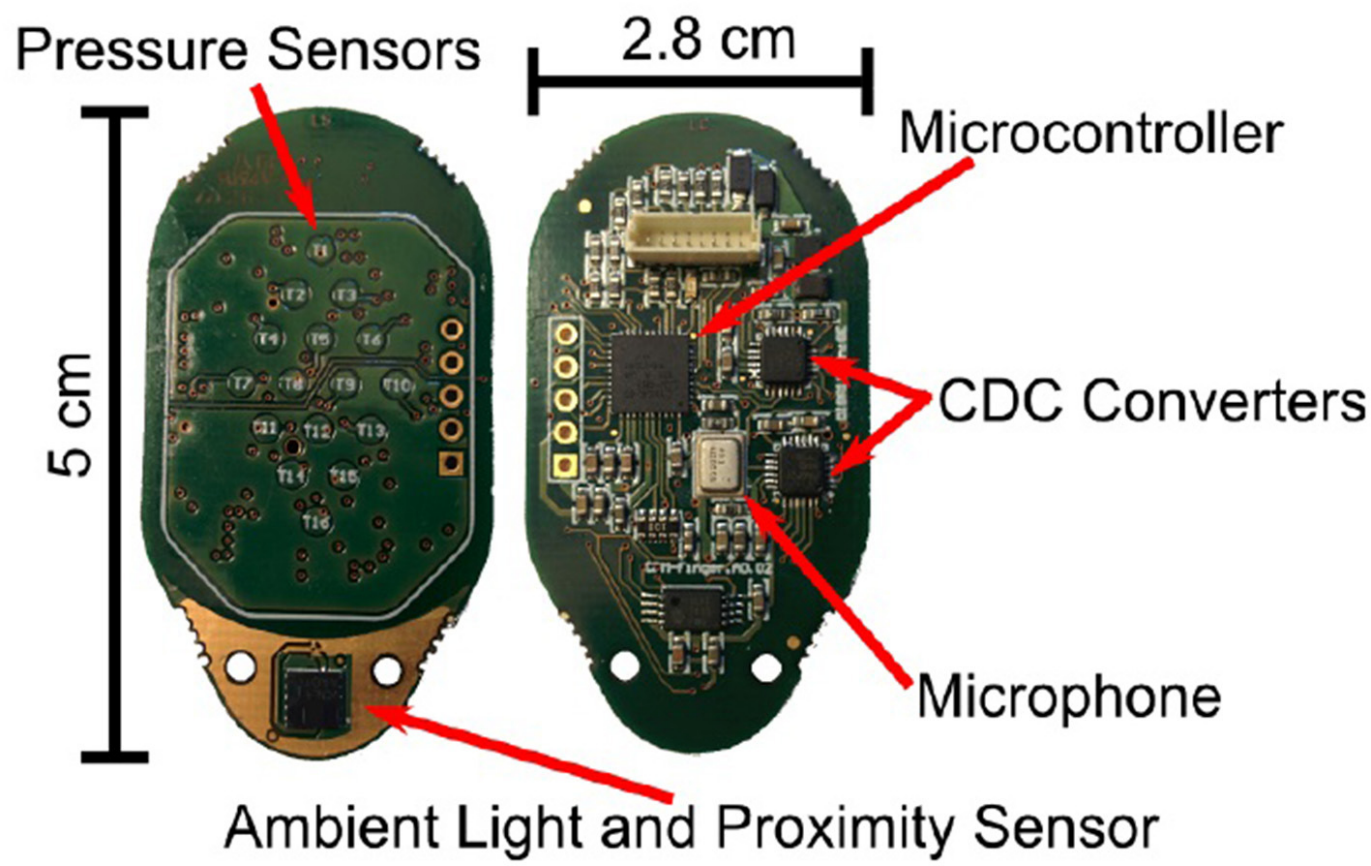
The top and bottom view of the sensor circuit board. The side with the pressure sensors is in contact with the objects during experiments.

**Figure 3:**
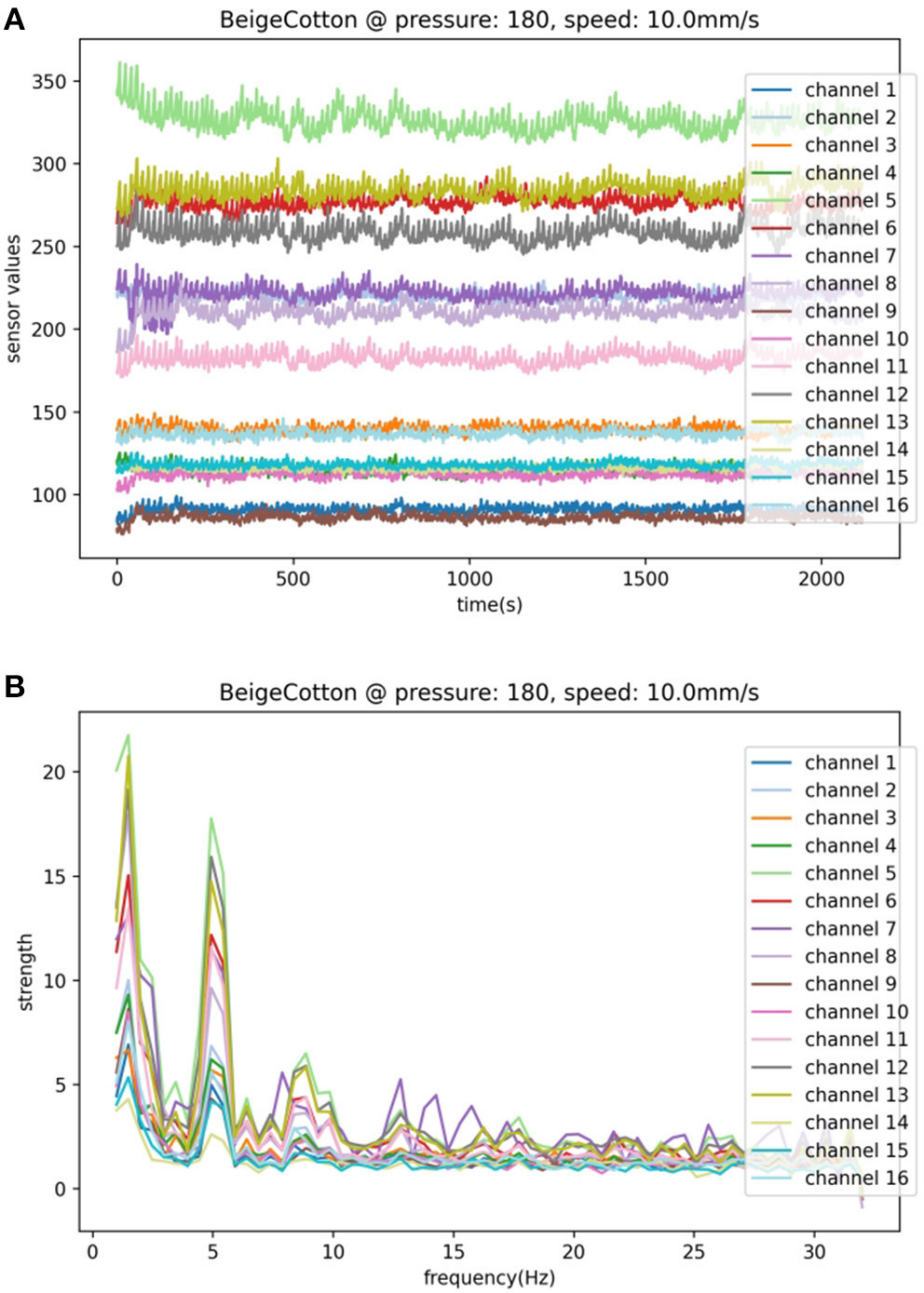
A sample of the multichannel tactile signals in **(A)** space domain and **(B)** frequency domain.

Our research employs a capacitive tactile sensor CPM-Finger (outcome of the European Project CloPeMa - Clothes Perception and Manipulation, shown in Figure 1) introduced in Denei et al. (2017), developed for fabric detection and manipulation. It collects the vibration during the interaction with the object. Compared to other common types of tactile sensors including piezoelectric/piezoresistive sensors, triboelectric sensors, and optic sensors, the capacitor-based sensor has a wider dynamic range and is more robust, suitable for scanning (or sliding on) objects and its compact size allows easy integration into most robotic systems (Nicholls and Lee, 1989; Al-Handarish et al., 2020). The sensor contains 16 small capacitors (shown in Figure 2), i.e., taxels (as pixels for the visual sensor) that convert physical deformation of the elastomer to the variation of capacitance. The contact surface is covered by Spandex as a protective fabric. The sensor is based on the fact that, for a parallel-plate capacitor, the capacitance can be described by


(13) $$\begin{array}{l} C=\epsilon \frac{A}{d\left(P\right)} \end{array}$$

where ϵ is the permittivity of the dielectric middle layer, *A* is the overlap area of two parallel plates, and *d*(*P*) is the distance between the two plates as a function of the applied pressure *P*. At the sampling rate of 32 Hz, the sensor signals in 1 s can be arranged into a matrix $X\;\in \;R^{32\times 16}$ (shown in Figure 3 for an example). For each capacitor, there is a baseline value output from the capacitance-to-digital converter (CDC) at zero pressure. The value has been subtracted from the sensor reading at the firmware level such that the output sensor signals share the same value ranges and rest at 0 without pressure applied. For that reason, the sensor signals do not convey an exact physical meaning and we can comfortably omit the unit μ*F* and carry the values around for simplicity.

### 4.2. Robotic Sliding Experiments

The sliding experiments are implemented with a Franka Emika Panda 7DOF robotic arm with a two-finger gripper as the end-effector. One CPM-Finger tactile sensor is installed on the gripper to replace the original rubber fingertip.

**Figure 4:**
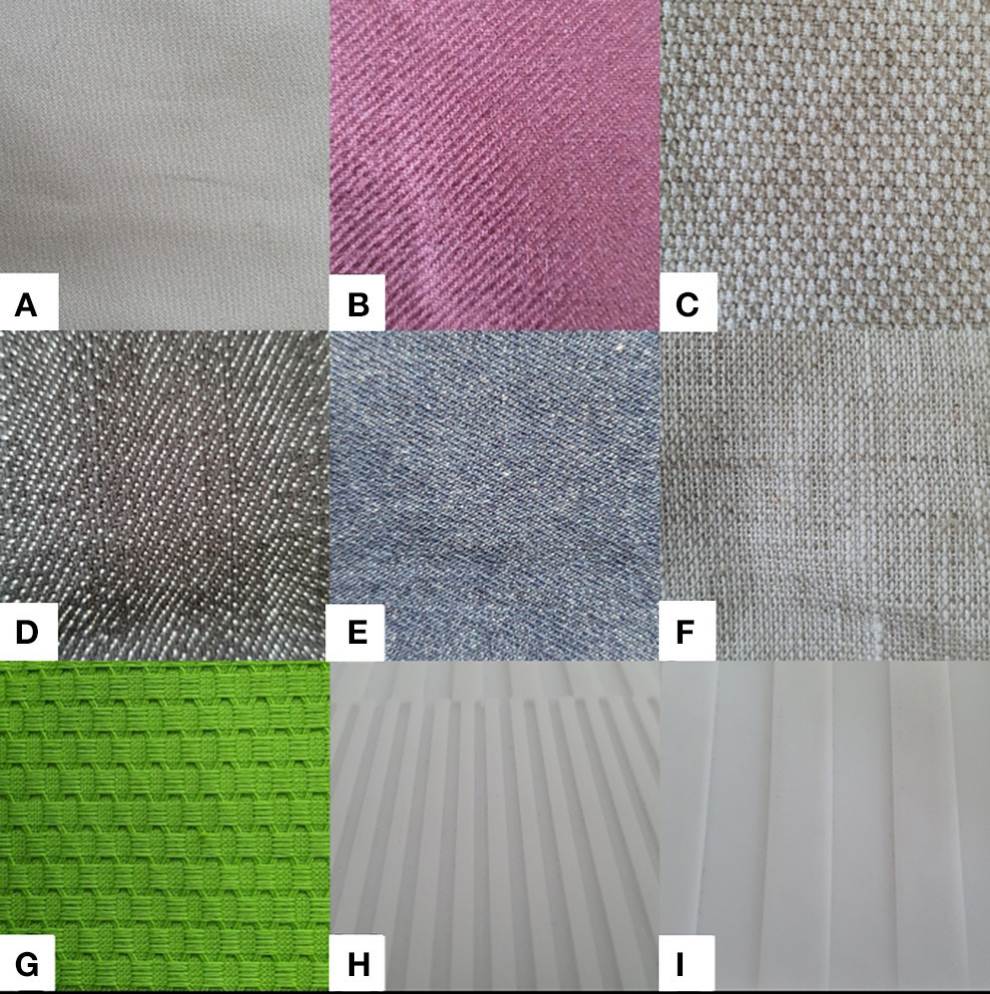
Fabric samples **(A)** *BeigeCotton*, **(B)** *BrownCotton*, **(C)** *Canvas*, **(D)** *Denim*, **(E)** *DenimFlex*, **(F)** *Linen*, **(G)** *WovenFabric*, **(H)** *Board2mm*, and **(I)** *Board10mm*. Labels of the fabrics are only for identification in the experiments and are not related to the exact material or any trademark.

**Figure 5:**
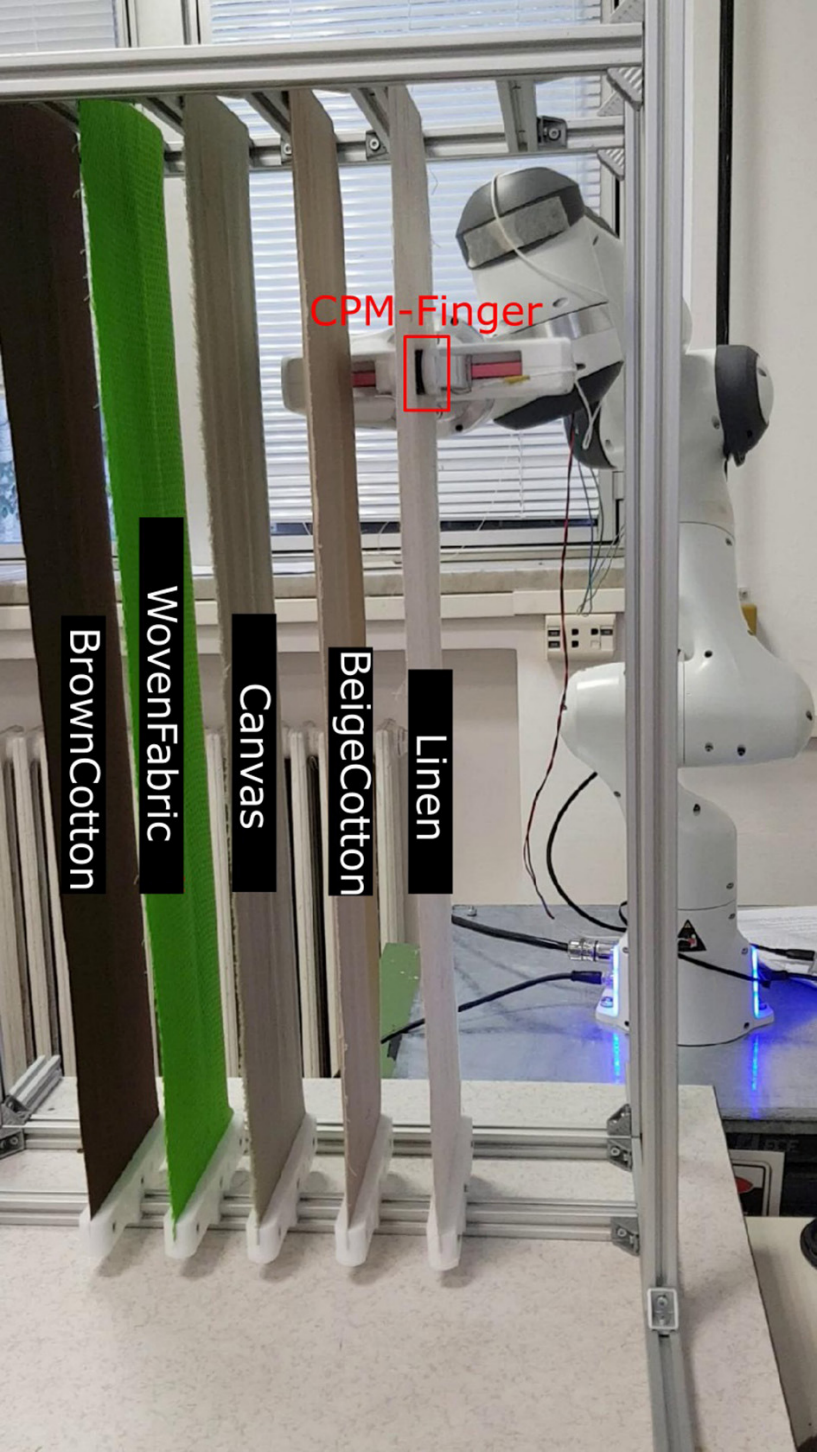
Illustration of the experimental setup. The original rubber fingertip of the gripper is replaced with a CPM-finger sensor. Five fabrics are fixed on an aluminum rack at one time.

In total, seven types of fabrics are used for the experiments. We name them as *BeigeCotton, BrownCotton, Linen, Canvas, Denim, DenimFlex*, and *WovenFabric* as shown in Figure 4. They are cropped into 65 cm × 10 cm stripes with both ends clipped to an aluminum rack. Fabrics are tensioned roughly to guarantee a vertical positioning (shown in Figure 5), but not over-stretched so that they can still be extended and twisted during the robotic sliding motion. Precise measurement of the fabric tension is beyond the scope of our experiments due to the following considerations:

1. It is not always possible to measure the exact tension of the fabrics in real applications given the complex forms of the fabrics;
2. Non-uniform tension among the fabrics can serve as a testimony of robustness of our methods;
3. Due to the friction between the protective fabric Spandex and the inspectee fabric, stretching and twisting happen during sliding in a hard-to-predict way, preset tension has a little indication to the results, especially for higher sliding pressure settings.

The robot gripper is controlled to grip the fabric stripe with constant pressure and slide vertically at a constant speed to collect one set (3 samples a set) of tactile signals for each parameter setting (sliding up and down in the same velocity are considered as two speeds). The grip pressure is maintained *via* proportional–integral–derivative (PID) control on the closing distance between the fingertips. We capture the average value of the 16 sensor readings as an *indicator of the grip pressure (Pressure)*.

The control command is computed and sent to the robot host controller through a Linux OS patched with a realtime kernel.

### 4.3. Fabric Slide Dataset

The data acquisition proceeds as follows:

1. The robot closes the gripper till the desired *Pressure* is reached, and holds the *Pressure*.
2. The robot moves the gripper vertically with a constant speed downward and then upward for a distance of 50 cm, respectively, at the same velocity. The tactile signals as time series are captured during the process and stored in the format of a matrix.
3. Each pair of sliding parameter settings is repeated 3 times. Then, the robot releases the gripper, moves horizontally away from the current tested fabric stripes, and shifts to the next fabric, till all fabrics are tested with the current sliding speed and holding pressure.
4. The robot repeats the whole sliding experiments from steps 1 to 3 with different pairs of control parameters, i.e., speeds and *Pressure*, as listed in Table 1, on all fabrics.

**Table 1:** Combinations of sliding parameters are chosen from this table.

**Sliding Parameters**	
**Speed (mm/s)**	**Pressure**
10	120
20	150
50	180
100	210
120	250
150	N/A

Two types of denim fabrics, i.e., *Denim* and *DenimFlex* in our nomenclature, skip through the sliding with *Pressure* 250, and *WovenFabric* passes through both *Pressure* 210 and 250, as in these cases, the torques required to conduct the sliding motions exceed the maximum payload of the robot due to severe folding and twisting of the inspectee fabrics caused by frictional force. With all the other available *Pressure* settings, sliding motions in all 6 speeds are executed. In total, 966 samples are collected as matrices in the shapes of *N* × *M* where *N* is dependent on the duration of the sliding motion and *M* is the number of signal channels, i.e., the number of taxels, which is 16 for our CPM-finger sensor.

## 5. Analyses and Results

To simulate the scenario where new fabric classes are presented, we follow an iterative process to update the feature extractor and test the classifier:

1. We first randomly select two fabrics, e.g., *Canvas* and *DenimFlex* as prior knowledge, i.e., initial training classes, to fit the IPCA feature extractor.
2. The measurements from the two training classes are transformed into the feature space by the feature extractor just trained on them.
3. Tactile measurements of all the other (unfitted) fabric classes serve as the test dataset. They are transformed into feature vectors to testify to the classifier.
4. Randomly pick one unfitted fabric class as a newly presented class to update the IPCA feature extractor with a partial fitting method. Add the class to the training classes.
5. Project the data of training classes into the feature space.
6. Repeat from 3.

**Figure 6:**
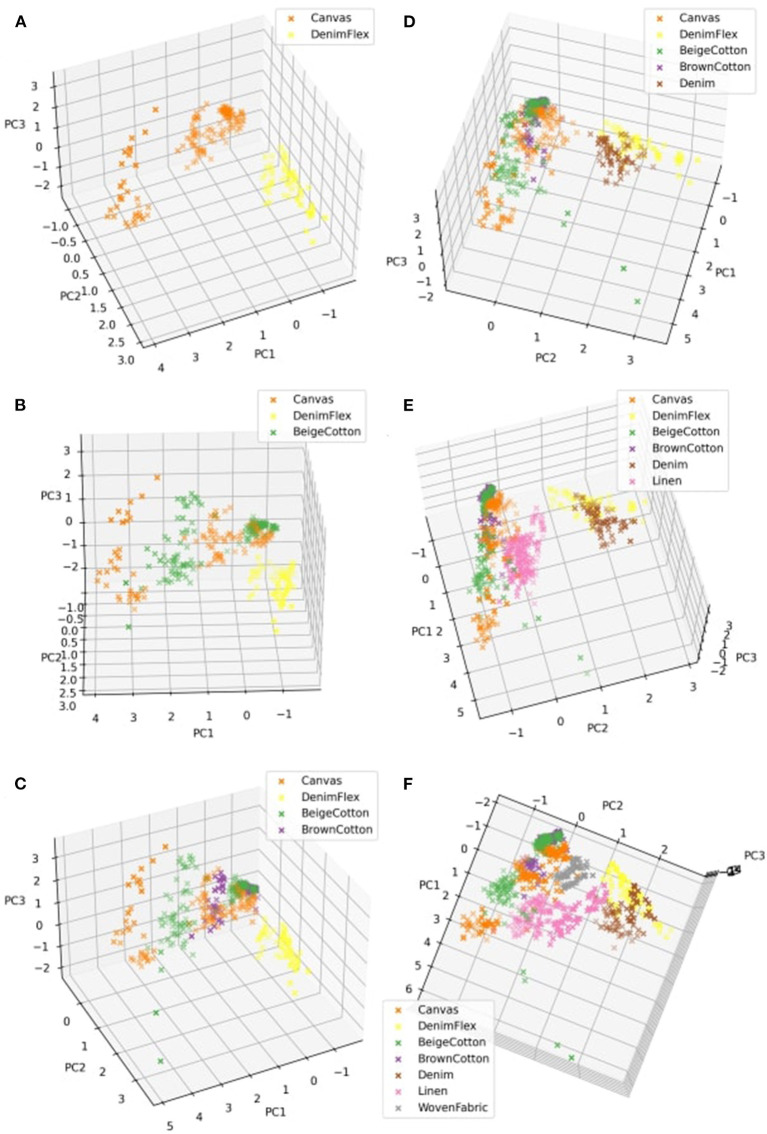
3D feature space of the tactile signals acquired sliding trials. **(A–F)** correspond to feature spaces of 2 to 7 fabrics in the process of iterative feature extraction.

The feature space and the projected data points of our randomly selected training classes, *Canvas* and *DenimFlex*, in the 3D space (shown in Figure 6A). Two fabric classes separate apparently, very likely due to their intrinsic difference in textures, elasticity, and friction, which can also be perceived and discerned with human touch with ease.

Then, a new fabric *BeigeCotton* is presented as a testing class. The tactile measurements of the first test class are transformed to 3D feature vectors by the IPCA feature extractor trained solely on the first two **training classes**, to join the feature space where 3 fabrics are presented now (see Figure 6B). The first test fabric *BeigeCotton* intertwines with *Canvas* in the feature space as they are both in plain knit. The minor resemblance in textures puts them in a similar region in the feature space. After observing the visualization of our new 3-class feature space, we reuse the original tactile measurements of *BeigeCotton* to partially fit our incremental IPCA feature extractor. In the presence of the next new fabric, the feature extractor has already been updated to include the fabric class *BeigeCotton*.

Similarly, Figures 6C–F show feature spaces associated with the incremental process of incorporating more testing fabrics to the IPCA feature extractor. The visualization of the feature space gives a hint that *BrownCotton* and *BeigeCotton* can be hard to distinguish under some circumstances; *Canvas* lies in the large common region of other *Cotton* but it responds to different pressure settings in its own way that deviates from *BeigeCotton* and *BrownCotton*. *Linen* demonstrates some essentially different features that stand out from other fabrics. The elasticity is much greater than *Cotton* and *Canvas*. While the textures on the surface are not as smooth and even as other fabrics, it could be the reason that *Linen* scatters irregularly in the feature space. The embrace of *Denim* and *DenimFlex* in the feature space is consistent with the similarity of the two types of *Denim* in textures and elasticity, which again can be verified by human touch.

**Figure 7:**
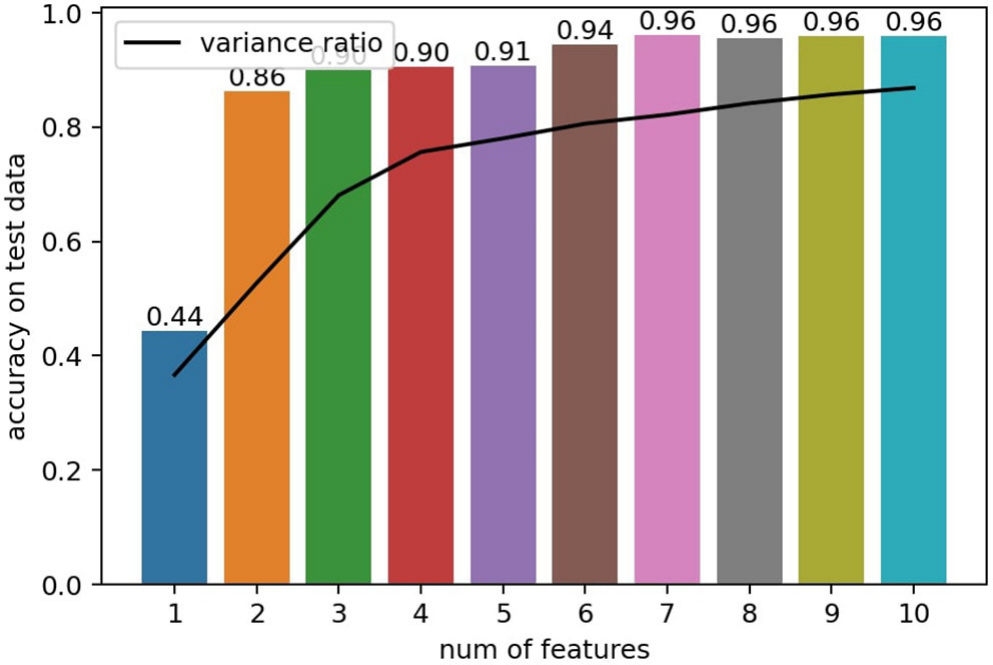
Number of (incremental principal component analysis, IPCA) features vs. Classification accuracy of a k-NN classifier, with *k* = 10 using half of the dataset as a test set. The black line follows the ratio of variance explained by the principal components.

With all the tactile measurements projected into the feature space, we split the feature vectors of all classes in halves as one training dataset and one testing dataset to fit and test a k-NN classifier taking *k* = 10. First, we show how the number of features *D* extracted by the IPCA method affects the classification performance on the testing set (shown in Figure 7). Trends of classification accuracy are congruent with the change in the ratio of variance explained by *D* principal components. When *D* ≥ 7 the classifier reaches its limit in our experiments, where classification performance ceases to improve.

**Figure 8:**
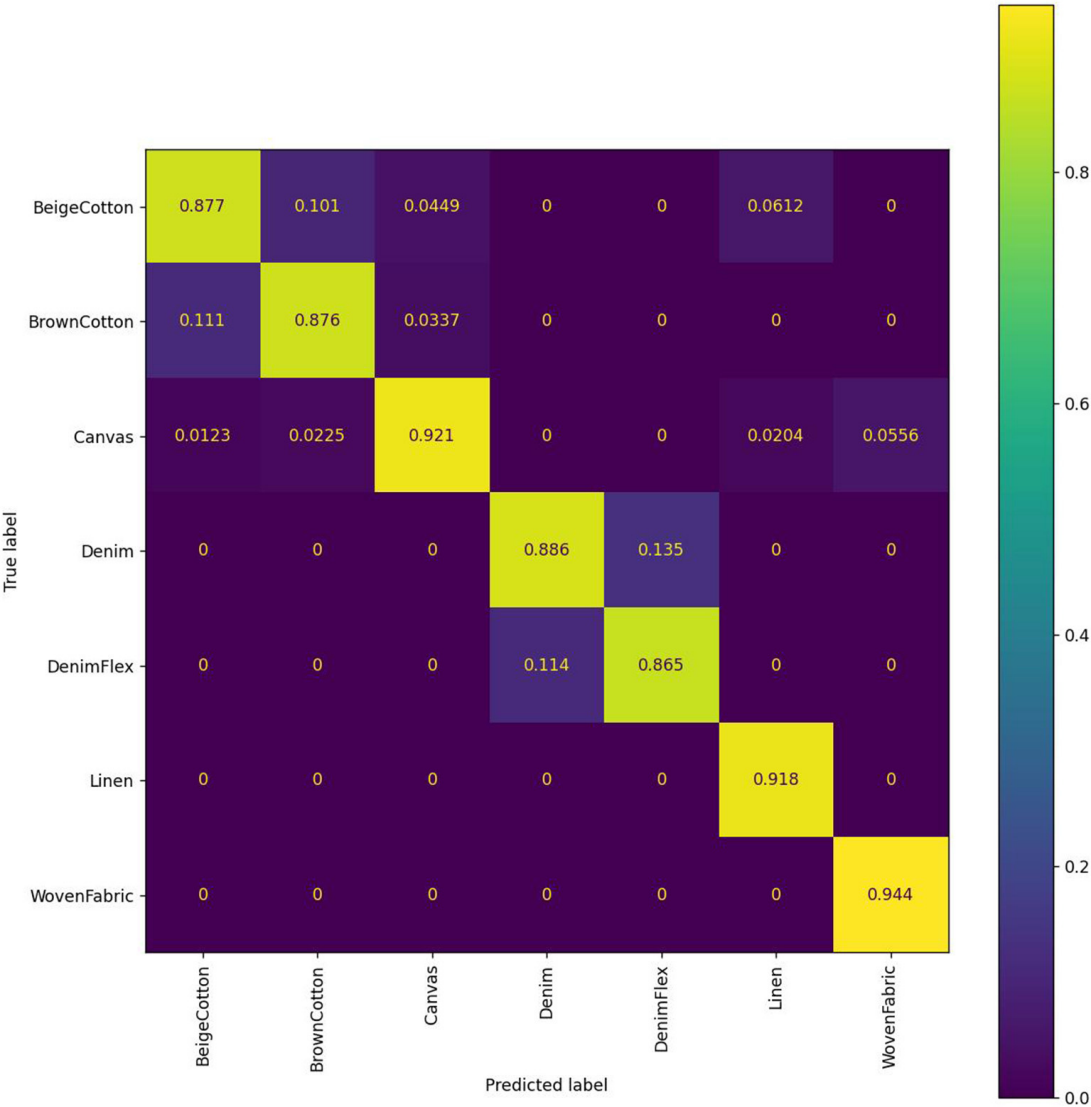
Confusion matrix of 7-fabric classification using a k-NN classifier with 3 IPCA features.

To be consistent with all the 3D visualization, we use a feature extractor of *D* = 3 principal components (PCs). We first show the confusion matrix (shown in Figure 8) using half of the samples as a training dataset and the other half as a testing dataset, to have a rough picture of the classification performance. Entries with higher confusion rates are well matched to the fabrics classes that are tangled in the feature space in Figure 6F.

**Figure 9:**
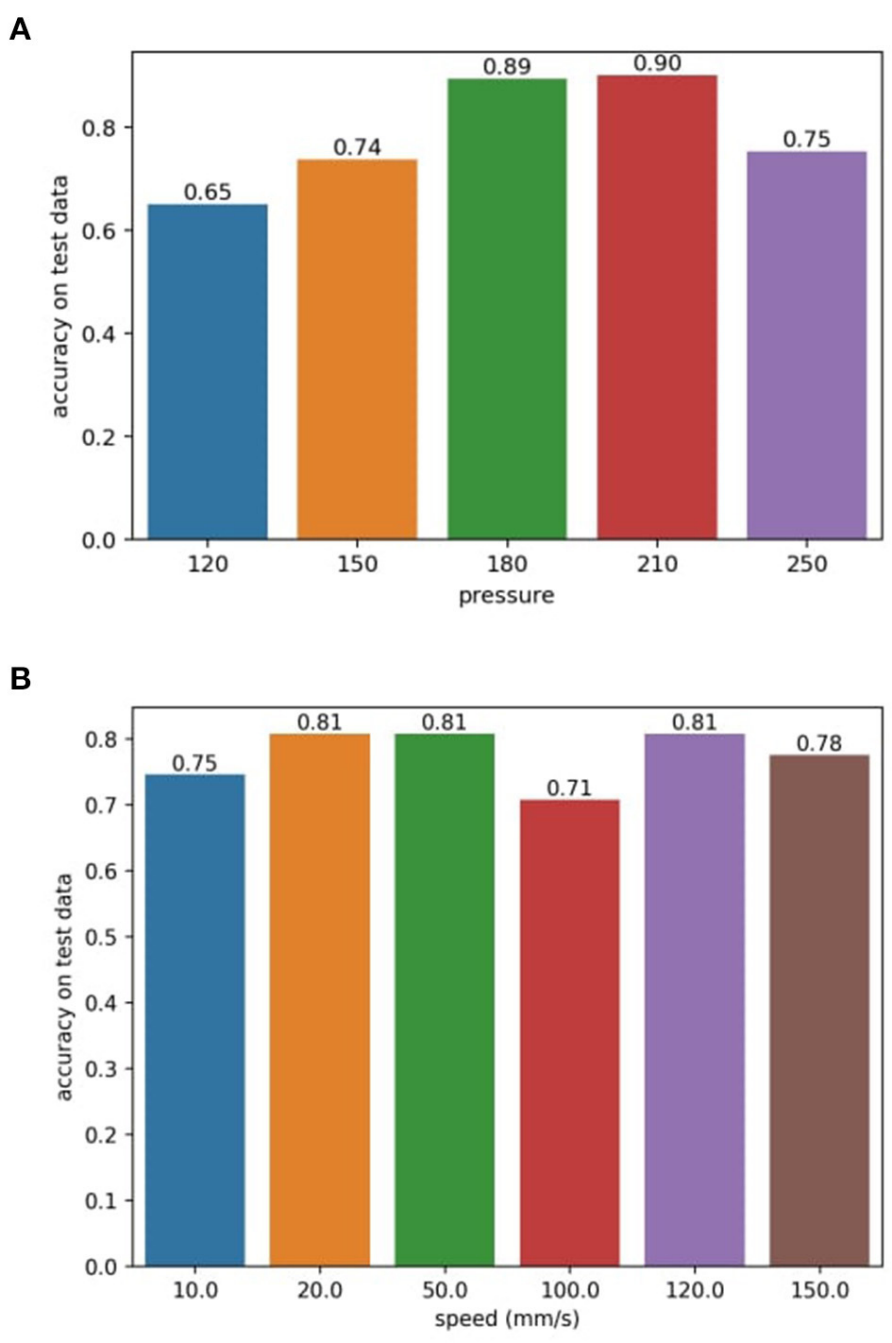
**(A)** Sliding pressure (as the average value of the 16 taxel signals, unitless) vs. Classification accuracy **(B)** Sliding speed vs. Classification accuracy.

**Figure 10:**
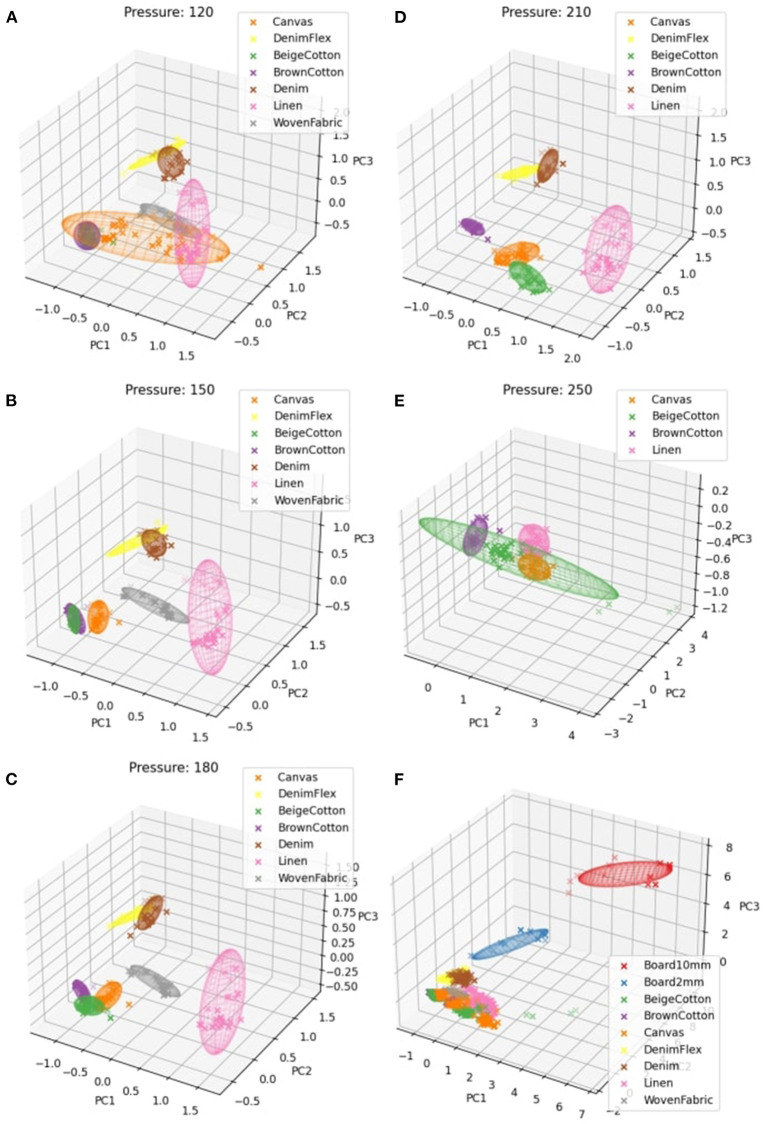
Feature spaces **(A–E)** correspond to sliding pressure settings of 120, 150, 180, 210, and 250 of only fabrics. **(F)** is the feature space of all fabrics and polylactic acid (PLA) boards with all different speed and pressure settings. The 95% confidence ellipsoids are shown to illustrate the intra-class dispersion.

All the above results combined feature vectors sampled with all different sliding parameters specified in Table 1. To check how the fabric classes separate for each *Pressure* setting, we first show the feature spaces corresponding to only one *Pressure* (while speeds are still mixed) at a time. With the same feature extractor trained on *D* = 3 PCs, the classifier shows a performance fluctuation under different *Pressure* settings (see Figure 9A). The sweet spots fall at *Pressure* 180 and *Pressure* 210 where the classifier shows significantly better performance. The results coincide with the better segregation of fabric clusters as in Figures 10C,D. We also show the 95% confidence ellipsoids to help visualize the change of clustering along with the pressure change.

Since only 4 fabrics can be sampled under *Pressure* 250, the results shown in Figure 10E is only for reference without being directly comparable to other pressure settings. Hence, it is plausible to infer that higher holding pressure contributes to better classification of fabrics in the sliding motion. Moreover, the slightly better yet negligible improvement in classification in the range of pressures from *Pressure* 180 to 210 indicates a potential saturation of grip pressure, which refers to a sufficiently (maybe fully) stretched or even over-stretched condition of the fabric stripes, where fabrics textures are severely distorted or even flattened out. Whether a fabric stripe is stretched enough for identification and classification might as well be closely related to the resolution and sensitivity of the tactile sensor itself. As can be seen in Figure 10A, data points of different classes remain at close distances from each other, which implies that under low pressures the extracted features carry insufficient information of the fabric classes. Increasing pressure to 150 significantly improves the discriminability (see Figure 10B) where only two types of cotton remain tangled. The two cotton stripes are very likely to differ subtly in the weaving method, making them tricky to distinguish. While for other fabrics, the slightly increased pressure already ensures sufficient interaction between the sensor and the textural surface to expose differences in the frequency domain.

**Figure 11:**
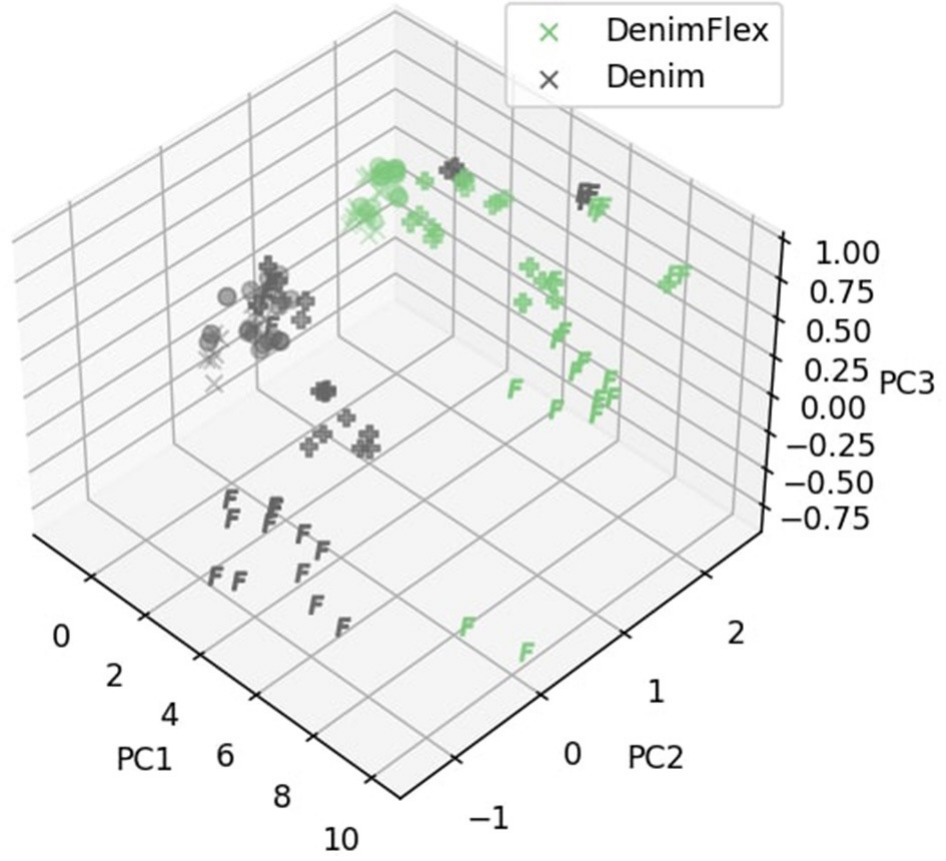
Samples of *Denim* and *DenimFlex* in the feature space. Markers of ×, ●, ✚ and **F** correspond to sliding pressures 120, 150, 180, and 210, respectively.

To better illustrate the effect of grip pressure, we show in Figure 11 data points sampled under different pressures with marks ×, ●, ✚, and **F** sequentially, in the ascending order. Data points are more scattered under larger pressures, which confirm our conclusion in the last paragraph that larger holding pressures help to extract more information from the fabrics. However, another notable phenomenon is that features are also less *consistent* (more scattered) under larger pressures. This is very likely caused by the very strong interaction between the sensor and the fabric stripes, where the sliding motion is not as smooth as it is under lower pressure settings due to augmented frictional forces. In the experiments, due to gravity, anisotropic “fingerprint” of the sensor and fabric folding and shifting, the sensor stutters during the sliding motion.

Finally, we show the effect of different sliding speeds on classification in Figure 9B. Classifications are conducted with a one-speed setting (only the magnitudes of speeds are considered). Sliding speeds are seemingly irrelevant to the features extracted under our experimental setup. Increasing or decreasing the sliding speed alone shows no major impact on the classification performance.

Given the results shown above, mostly the visualization of the feature space with clusters and the analysis of classifier performance concerning the parameters of sliding motions, we make a statistically sound inference that a low dimensional (circa ≤ 7D) vector is sufficient to feature the tactile measurement of textures sampled from our CPM-Finger tactile sensor. The feature vector is essentially a condensed form of the frequency spectrum which not only represents the frequency *signature* of a fabric texture but also embeds some characteristics of the tactile sensor itself. Even at a relatively low sampling frequency of 32 Hz, we can still reach a considerable classification accuracy of 96% for 7 fabrics by using a 7D feature extractor.

The fact that the same processing pipeline has significantly different performance on fabric classification when varying sliding motion parameters, supports our assertion (see Section 1) that, tactile sensing as a contact perceptive technique, is conceptually very different from the visual and auditory perceptions. Tactile sensors capture the information in the interaction with the environment, during which the interactive modes (i.e., the relative motion between tactile sensors and the objects in contact) and parameters are also reshaping the environment, that reversely affect the tactile measurement itself. Whereas for visual and auditory perceptions, movements of the sensory system are not mandatory to acquire signals and have no direct impact on the observable most of the time.

For a specific sensing technology, varying the parameters of the exploratory motions not only serves to enlarge the perceptive field and gain more information but also helps to seek the best interactive conditions of the tactile sensors regarding the object. The essence of tactile sensing is a capture of the generated deformation during the mechanical interaction between the sensor and the object surface. Changing the motion parameters for better classification performance can be viewed as a robotic adaptation to maximize the efficacy of the sensors and the perception system, given that specs of the sensors (e.g., sampling rate, resolution) and the physical properties of the object are likely unalterable.

## 6. Discussion

In this study, we focus on the classification task of only fabrics. However, it is natural to question whether the same methods apply to the classification of general materials. Some preliminary results of the experiments on a 3D printed polylactic acid (PLA) board with two types of Boards (with 2 and 10 mm grilles, respectively, shown in Figures 4H,I) show that using the feature extractor proposed in Section 3.2, trained on all fabric samples, the tactile measurements of the PLA board can be transformed into the same feature space (shown in Figure 10F). It accordingly seems that our proposed methods may also be promising in classifying non-fabric materials. The first step to extend our research will be simply adding more materials in the forms that are suitable for the same sliding motions. In that case, we can reach a more comprehensive understanding of whether for capacitive tactile sensors, the frequency spectrum alone suffices to feature a general texture.

Readers may also argue that the k-NN classifier might not be the best performer in material classification in our problem. A comparison between different methods including artificial neural network classifiers, decision tree classifiers, naive Bayesian classifiers, etc., can bring a better idea of the classification accuracy. The fact that is presented in this study, beyond the results of classification itself, most importantly, is that even a single modality tactile sensor at a low sampling frequency is already capable of classifying fabric materials by using a simple sliding motion, to a reasonable good accuracy (90–96%) with no more than 7D feature vectors. Tactile measurements represented by points in the low dimensional frequency feature space are segregated by their fabric classes naturally. These clusters can possibly be depicted by parameters of probabilistic models, e.g., Gaussian mixture, to form a more compact representation of tactile knowledge base. This implies the great potential of tactile sensing in other tasks relating to object identification and classification.

## Data Availability Statement

The datasets presented in this study can be found in online repositories. The names of the repository/repositories and accession number can be found at: GitHub, https://github.com/wngfra/FabricSlide.

## Author Contributions

SW performed the work and contributed to the writing of the manuscript. AA contributed to the writing of the manuscript. GC provided research supervision, and contributed to the manuscript revision, and read and approved the submitted version. All authors contributed to the conception of the project.

## Funding

The research was entirely supported by the University of Genoa.

## Conflict of Interest

The authors declare that the research was conducted in the absence of any commercial or financial relationships that could be construed as a potential conflict of interest.

## Publisher's Note

All claims expressed in this article are solely those of the authors and do not necessarily represent those of their affiliated organizations, or those of the publisher, the editors and the reviewers. Any product that may be evaluated in this article, or claim that may be made by its manufacturer, is not guaranteed or endorsed by the publisher.
